# Enhanced Multiferroic Properties of YFeO_3_ by Doping with Bi^3+^

**DOI:** 10.3390/ma12132054

**Published:** 2019-06-26

**Authors:** Omar Rosales-González, Félix Sánchez-De Jesús, Fernando Pedro-García, Claudia Alicia Cortés-Escobedo, Màrius Ramírez-Cardona, Ana María Bolarín-Miró

**Affiliations:** 1 Área Académica de Ciencias de la Tierra y Materiales, Universidad Autónoma del Estado de Hidalgo Mineral de la Reforma, Hidalgo 42184, Mexico; 2 Instituto Politécnico Nacional, Centro de Investigación e Innovación Tecnológica, Ciudad de Mexico 02250, Mexico

**Keywords:** YFeO_3_, Bismuth doped YFeO_3_, high-energy ball milling, ferromagnetic, dielectric, multiferroic

## Abstract

Tthe present work studied the cationic substitution of Y^3+^ by Bi^3+^ on the crystal structure of orthorhombic YFeO_3_ and its effect over magnetic, dielectric and electric properties of multiferroic yttrium orthoferrite. Stoichiometric mixtures of Y_2_O_3_, Fe_2_O_3_ and Bi_2_O_3_ were mixed and milled for 5 h using a ball to powder weight ratio of 10:1 by high-energy ball milling. The obtained powders were pressed at 1500 MPa and sintered at 700 °C for 2 h. The test samples were characterized at room temperature by X-ray diffraction (XRD), vibrating sample magnetometer (VSM), scanning electron microscope (SEM), energy dispersive X-ray spectroscopy (EDS) and impedance spectroscopy (IS). The X-ray diffraction patterns disclosed a maximum solubility of 30 % mol. of Bi^3+^ into the orthorhombic YFeO_3_. For higher concentrations, a transformation from orthorhombic to garnet structure was produced, obtaining partially Y_3_Fe_5_O_12_ phase. The substitution of Bi^3+^ in Y^3+^ sites promoted a distortion into the orthorhombic structure and modified Fe-O-Fe angles and octahedral tilt. In addition, it promoted a ferromagnetic (FM) order, which was attributed to both the crystal distortion and Dzyaloshinskii-Moriya interaction. For doped samples, an increase in real permittivity values was observed, and reduced with the increase of frequency. This in good agreement with the Maxwell-Wagner effect.

## 1. Introduction

Multiferroic materials are those that exhibit at least two of three ferroic orders: Ferroelectricity, ferromagnetism, and ferroelasticity [1]. Multiferroic that combines ferromagnetic and ferroelectric behaviors, also known as magnetoelectric multiferroic, is quite rare compared to other multiferroic materials., However, there has been considerable scientific interest for its potential applications in electronic systems, especially in storage devices for recording and reading information [2,3,4]. However, there are still many design challenges for multiferroics materials, highlighting that this behavior is exhibited only at low temperature [5]. Bismuth ferrite (BiFeO_3_) is the first generation of multiferroic materials that combine in a single phase ferroelectric and antiferromagnetic behavior at room temperature [6,7]. Furthermore, it presents many issues due to the formation of secondary phases that provide a high leakage current [8,9]. The second generation of multiferroic materials is composed by perovskites with the general formula RFeO_3_ where R = Y, Ho, Lu, Er or Sc. [10] YFeO_3_ is a second-generation multiferroic material. This material crystallizes in two polymorphic forms: (i) Orthorhombic perovskite-type structure; and (ii) hexagonal structure. The orthorhombic YFeO_3_ or yttrium orthoferrite, having distorted the perovskite structure, crystallizes in the centrosymmetric space group *Pnma*, with lattice parameters a = 5.5946 Å, b = 7.6040 Å, and c = 5.2790 Å. The centrosymmetric nature of this material is responsible for ferroelectric and weak ferromagnetic behaviors at room temperature. It presents a magnetic transition at the Neel temperature of ~640 K [11,12]. Magnetic ordering is related to three types of magnetic interactions in atoms bonds: Y-Y, Y-Fe and Fe-Fe where Fe-O-Fe super-exchange interaction is the main source of the weak ferromagnetic behavior as well as the canting of magnetic moments of Fe^3+^ due to the Dzyaloshinskii-Moriya (DM) interaction [10,13]. Further, centrosymmetric structures are not ferroelectric in nature, however distortions in the structure can modify it [14]. For YFeO_3_ materials, *Pnma* centrosymmetric structure induces polarization arising from canted antiferromagnetic ordering and structure distortion [15,16]. This indicates that multiferroic property of this material is associated with Fe-spins which induces electric polarization, partial substitution or doping with different ions in A or B sites (Y^3+^ and Fe^3+^ sites) which are an advantageous method to modify and sometimes enhance the multiferroic properties of YFeO_3_ [17,18]. Many works have been focused on the doping of one of both atomic positions reporting interesting results in multiferroic properties. Jacobs et al. reported increased electrical conductivity with the partial substitution of Ca^2+^ for Y^3+^ into YFeO_3_ crystal structure, which was attributed to the compensation of charge Ca^2+^ that produced a hole localization on Fe site [19]. The substitution on A-sites with magnetic ion Gd^3+^ enhanced the magnetic behavior mainly due to the new Gd-Gd, Gd-Fe interactions, structural distortions and microstrain induced in the YFeO_3_ crystal structure [20,21]. Similar behavior was observed for the doping of Er^3+^ substituting Y^3+^ at low concentrations. Nevertheless, when Er^3+^ content increased, the weak ferromagnetic behavior was lost and acquired paramagnetic properties [22]. A dielectric properties study on YFeO_3_, concerning to B site substitution of Fe^3+^ with Mn^3+^, showed a reduction in relative permittivity and three dielectric relaxations in the function of temperature. Further, the results showed the important role of oxygen vacancies toward the dielectric relaxation process [3,17]. Madolappa et al. [11] reported an increased coercive field of YFeO_3_ related with the use of Ti^4+^ as a doping ion. They demonstrated that the dielectric relaxation process corresponded to the non-Debye type. Additionally, Ti^4+^ promoted a long-range conductivity process. Regarding the above, R^3+^-Fe interactions demonstrated an intimal relation with the magnetic and dielectric properties of the orthorhombic YFeO_3_. Therefore, it is possible to modulate ferromagnetic and ferroelectric properties with the addition of rare earth cations with a different ionic size other than the Y^3+^ ionic ratio. It is expected that Bi^3+^ would enhance the ferroelectric behavior, in a similar way that occurs in BiFeO_3_, but avoiding the formation of secondary phases and increasing the FeO_6_ octahedron tilting, providing a ferromagnetic behavior. 

This work reports a systematical study of the partial substitution of Y^3+^ by Bi^3+^ to modulate the ferromagnetic and dielectric properties of orthorhombic YFeO_3_. The nature of the origin of ferromagnetic and dielectric properties has not been clearly explained yet, therefore it is important to evaluate individually the magnetic and the dielectric behaviors as indicators for its prospective application as multiferroic material.

## 2. Materials and Methods 

Stoichiometric mixtures of precursor oxides: Iron oxide (Fe_2_O_3_, Sigma-Aldrich, St. Louis, MO, USA, 99.9% purity), yttrium oxide (Y_2_O_3_, Sigma-Aldrich, St. Louis, MO, USA, 99.9% purity) and bismuth oxide (Bi_2_O_3_, Sigma-Aldrich, St. Louis, MO, USA, 99.9% purity) were mixed following to Equation (1):

(1) $${(1-x)Y}_{2}O_{3}{\;+\;\mathrm{Fe}}_{2}O_{3\;}{+\;x\;\mathrm{Bi}}_{2}O_{3}\to {\;2Y}_{1-x}{\mathrm{Bi}}_{x}{\mathrm{FeO}}_{3}$$

A total of 5 g of starting mixture was loaded along with steel balls of 1.27 cm of diameter in a cylindrical steel vial (50 cm^3^) (steel/steel, S/S) at room temperature in an air atmosphere and milled for 5 h, using a shaker mixer mill (SPEX model 8000D, SPEX^®^ SamplePrep, Metuchen, NJ, USA). The milled powders were uniaxially pressed into cylindrical test pieces at 1500 MPa. The test pieces were annealed in an air atmosphere at 700 °C for 2 h. The structural characterization of all the products was made using an X-ray diffractometer Bruker D-8 (Bruker Corporation, Billerica, MA, USA) with Cu radiation (λ = 1.541874 Å) in the 2-theta range from 20 to 60 degrees. Refined lattice parameters, crystallite sizes, phase quantification (wt. %) and microstrain (µε), were determined by Rietveld analysis using free-software, Material Analysis Using Diffraction (MAUD, Version 2.26, Trento, Italy)), for all synthesized samples. This method considers all the collected information in a diffraction pattern and uses a least-squares approach to refine the theoretical line profile until it matches the measured profile [23]. Starting crystallographic data were obtained from the Inorganic Crystal Structure Database (ICSD). The grain morphologies and porosity of sintered pellets were analyzed using a JEOL JSM-6300 scanning electron microscope (JEOL, Akishima, Tokio, Japan) working at an accelerating voltage of 15 kV. Energy-dispersive X-ray spectroscopy (EDS) microanalysis (EDAX, Mahwah, NJ, USA) was used to determine the elemental composition in SEM micrographs. Magnetization studies were carried out at room temperature using a vibrating sample magnetometer (VSM) model MicroSense EV7 (Microsense LLC, Lowell, MA, USA), with a maximum field of ±18 kOe. The Curie temperature was determinated by means of a temperature scan test under a magnetic field of 10 kOe and it was taken as the intersection point with the temperature axis of the tangent to the magnetization curve with the most negative slope. Impedance spectroscopy was performed using a LCR meter Model HIOKI Hi-TESTER, 3532-50 (HIOKI, Nagano, Japan) at room temperature, in a frequency range from 10^2^ to 5 × 10^6^ Hz. Previously, both sides of test pieces were coated with an Au-Pt alloy through the sputter coating process. 

## 3. Results and Discussions 

### 3.1. Structural Characterization 

**Figure 1:**
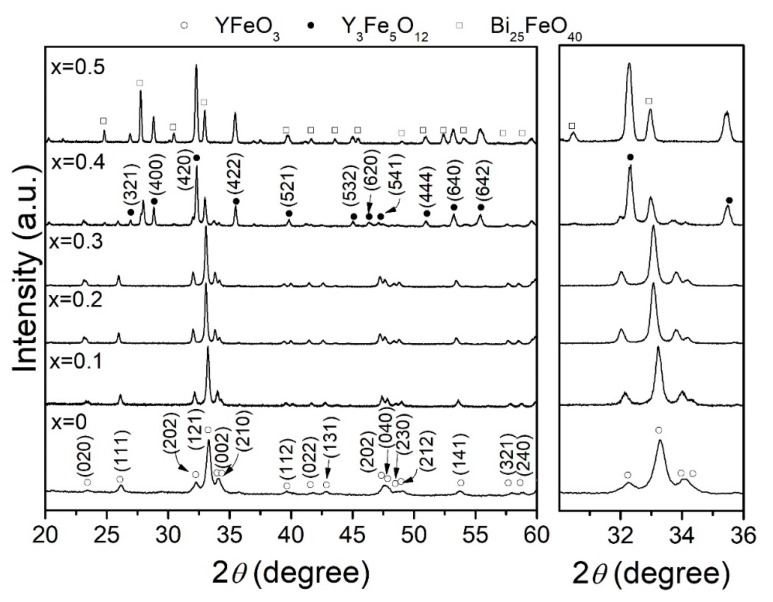
XRD patterns of samples of mixtures of Y_2_O_3_, Fe_2_O_3_ and Bi_2_O_3_ milled for 5 h and sintered at 700 °C for obtaining Y_1−x_Bi_x_FeO_3_ (0 ≤ x ≤ 0.5, Δx = 0.1).

Figure 1 shows the X-ray diffraction (XRD) patterns for different stoichiometric mixtures of Y_2_O_3_, Fe_2_O_3_ and Bi_2_O_3_, milled for 5 h, pressed at 1500 MPa and sintered at 700 °C for 2 h in order to obtain Y_1−x_Bi_x_FeO_3_ test pieces (Δx = 0.1, 0 ≤ x ≤ 0.5). As can be observed in Figure 1, the X-ray diffraction pattern belonging to the un-doped Y_1−x_Bi_x_FeO_3_ sample (x = 0), the formation of orthorhombic YFeO_3_ (YFeO_3_, ICSD #80865, *Pnma*) can be confirmed by the presence of its diffraction peaks. After doping with low contents of bismuth (x ≤ 0.3), two gradual phenomena could be observed. The decreased peak diffraction broadened in relation with the increased amounts of Bi^3+^ concentration, and shifted toward to low angles of the diffraction peaks, as shown on the right of Figure 1. It is a consequence of Y^3+^ substitution by the Bi^3+^ into the yttrium orthoferrite crystal structure which increased their lattice parameters due to their larger size radii ion. By increasing the bismuth concentration (x ≥ 0.4), new diffraction peaks belonging to yttrium iron garnet (YIG, Y_3_Fe_5_O_12_, ICSD #60167, *Ia3d*) appeared, and it was accompanied with sillenite (Bi_25_FeO_40_, ICSD #68627, *I-23*) as a secondary phase. 

The phase transformation or decomposition of yttrium orthoferrite was completed for x = 0.5, and it was induced due to the YFeO_3_ orthorhombic phase which was an intermetallic compound, with a stoichiometric composition. Therefore, it just introduced small amounts of dopants. In the studied compositions, a maximum amount of 0.3 mol. of bismuth can be introduced into the yttrium position of the orthorhombic crystal structure (solubility), in good agreement with Van Hook’s phase diagram [24,25]. In general, it was observed that a decrement in broadening peak reflections with the increment of doped level (x), was associated with modifications in the crystallite size and microstrain.

Rietveld refinements were carried out to study the evolution of the cell parameters, crystallite size, microstrain, and phase quantification as the Bi^3+^ concentration (x) was increased. The results are presented in Table 1. Through phase quantification, it possibly confirmed that samples with low Bi^3+^ concentrations (x ≤ 0.3) were mainly composed of pure orthorhombic YFeO_3_. However, when the bismuth was higher than 0.4, the amount of orthorhombic YFeO_3_ decreased until 15.97 wt. %, with an increment of the yttrium iron garnet (YIG or Y_3_Fe_5_O_12_) approximately 73.67 in wt. %, and 10.36 wt. % of Bi_25_FeO_40_. Moreover, for x = 0.5 sample, a complete phase transition to the garnet structure was confirmed, though Bi_25_FeO_40_ (13.32 wt. %) as a secondary phase was still present. In reference with refined parameters, it can be observed that the lattice parameters increased systematically, while the microstrain decreased as bismuth content increased. This can be attributed to the substitution of Y^3+^ with smaller ionic radii (0.90 Å) by a larger ionic radii cation Bi^3+^ (1.03 Å) [26]. Also, the microstrain can be correlated with the increased crystallite size and hence with the grain growth which promoted the liberation of internal stress of the crystal structure. These results explain the diminution of the broadening of peaks with the bismuth content, observed qualitatively in the XRD patterns shown in Figure 1. In the particular composition of x = 0.4, the polymorphic transition from orthorhombic YFeO_3_ to garnet Y_3_Fe_5_O_12_ produced a diminution in microstrain, independently to crystallite size. It was attributed to the preference of Bi^3+^ to form Bi_25_FeO_40_; rather than introduce into the crystal structure to obtain Bi doped YFeO_3_. The refinement parameters exhibited a good adjustment, as the low values of R_wp_ and χ^2^ have shown.

Geometrical stability of a perovskite-type phase can be explained by the Goldschmidt tolerance factor (t) [27]. This parameter correlates the symmetry system directly with properties in perovskite-type materials. The tolerance factor value is commonly in the range from 0.80 to 1.10 for perovskites, and especially, for orthorhombic structures which take values in the range of 0.8 to 1 [28]. The Goldschmidt tolerance factor (t) was calculated using the Equation (2) where r_Bi_ and r_Y_ corresponded to the ionic ratio of Bi^3+^ and Y^3+^ in 6 coordination while r_Fe_ and r_O_ corresponded to Fe and O ions. The data were obtained from the Shannon ionic radii [26]. The results are presented in Table 2.

(2) $$t\;=\;\frac{\left(\left(1-x\right)r_{Y}\;+\;x\;r_{\mathrm{Bi}}\right){+r}_{O}}{\sqrt{2}{(r}_{\mathrm{Fe}}{+r}_{O})}$$

In orthorhombic YFeO_3_ crystal structure, Fe^3+^ ions were surrounded by six O^2−^ which placed an octahedral (FeO_6_) where O^2−^ ions were located at the corner and shared between two octahedral structures providing two kinds Fe-O-Fe super-exchange bonds. Therefore, there were two bond angles (θ1 and θ2 for Fe-O1-Fe and Fe-O2-Fe, respectively). A geometrical relation between θ1 and θ2 angles and octahedral tilts (φ_1_ and φ_2_) were predicted for yttrium orthoferrite using O’Keefe geometrical approximations [29] (Equations (3) and (4)) giving values of angle tilt which is a way to determinate a distortion of the octahedral structure.

(3) $${\theta 1=\cos}^{-1}\left|\frac{{2\;-\;5\cos}^{2}\varphi_{1}}{{2\;+\cos}^{2}\varphi_{1}}\right|$$

(4) $${\theta 2=\cos}^{-1}\left|\frac{{1-4\cos}^{2}\varphi_{2}}{3}\right|$$

Magnetic and ferroelectric properties in orthoferrites are strongly dependent of the octahedral tilts of FeO_6,_ as well as the Fe-O-Fe bond angle, that are important parameters to know. Crystallographic data obtained from Rietveld analysis were represented in Vesta (Visualization for Electronics and Structural Analysis) software [30], in order to determine bond angles θ1 and θ2 of bismuth doped YFeO_3_ (Y_1−x_Bi_x_FeO_3_), to calculate the octahedral tilts using Equation (3) and Equation (4). It is important to note that it only can be applied for samples with the orthorhombic crystal structure, that is, samples doped with x equal/lower than 0.3—in this range of doping level, the phase is orthoferrite. The results are presented in Table 2. As can be observed in the calculated data from the XRD patterns, atomic positions of oxygen changed when bismuth content increased. This displacement of oxygen atoms originated a change in two super-exchange bonds angles (i.e., θ1 and θ2) and, consequently, octahedron tilts can be observed from φ_1_ and φ_2_ angles.

**Figure 2:**
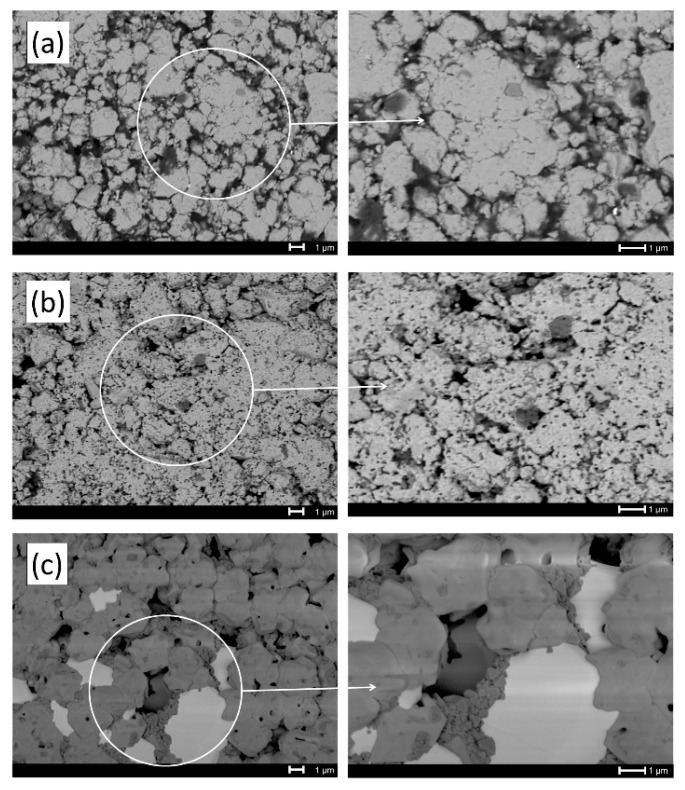
Scanning electron micrographs of samples: (**a**) x = 0, (**b**) x = 0.3 and (**c**) x = 0.5 annealed at 700 °C.

Backscattered electrons micrographs of Y_1−x_Bi_x_FeO_3_ doped at (a) x = 0, (b) x = 0.3 and (c) x = 0.5 sintered at 700 °C are showed in Figure 2. For sample x = 0 (Figure 2a), it was observed that grain size was approximately ~2 µm. This sample showed two regions with different color tonality associated to materials with different electron density, indicating the presence of two different phases. EDS analysis was carried out on both regions, founding the elements related to phases YFeO_3_ and Fe_2_O_3_ for white grains and small gray grains, respectively. The absence of Fe_2_O_3_ diffraction peaks in XDR analysis can be explained because their content is under the detection limit of the equipment employed. When x = 0.3 (Figure 2b), larger grain size was observed. This sample showed less porosity and minor porosity size. Also, still present the same phases as an un-doping sample where yttrium orthoferrite (white area) was a majoritarian phase with low quantities of hematite (grey areas). As well as the previous samples, for the sample with x = 0.5 (Figure 2c), only two phases can be observed. Nevertheless, EDS analysis indicated that in this case, the dark-grey regions corresponded to YIG phase and the white one corresponded to sillenite. An increase in grain size was observed in comparison with sample x = 0.3, and the porosity was reduced.

### 3.2. Magnetic Behavior

**Figure 3:**
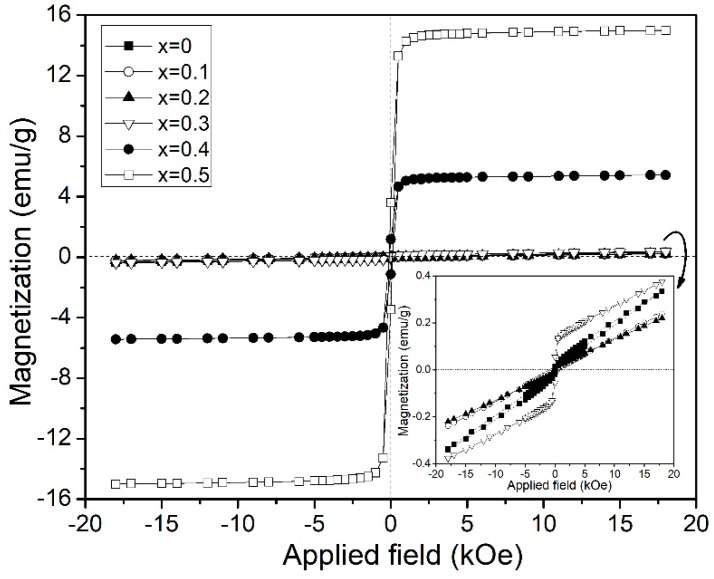
Magnetic hysteresis loops at room temperature of Y_1−x_Bi_x_FeO_3_ (0 ≤ x ≤ 0.5 ∆x = 0.1) sintered at 700 °C samples. The inset shows a magnification of hysteresis for 0 ≤ x ≤ 0.3 samples.

Figure 3 shows the magnetic hysteresis loops of the different stoichiometric mixtures of Y_2_O_3_, Fe_2_O_3_ and Bi_2_O_3_ to obtain Y_1−x_Bi_x_FeO_3_ varying x, from 0 to 0.5. As can be observed, the un-doped YFeO_3_ (x = 0) mostly showed an antiferromagnetic (AF) G-type order, that is, for each Fe^3+^ with a spin-up, the nearest Fe^3+^ neighbor possessed a spin-down. However, a weak ferromagnetic behavior can be observed for the un-doped sample, which was attributed to the presence of small amounts of Fe_2_O_3_., It was not detected by XRD analysis (under the limit of detection). Similar AF behavior was observed for samples doped with low Bi^3+^ contents (x ≤ 0.2), concluding that the substitution of Y^3+^ by Bi^3+^ cations did not promote important structural distortions which could modify the magnetic interactions. However, at low bismuth levels, it can be observed that a decrease of the specific magnetization at 18 kOe, with the increase of bismuth content, adscribed to the structure distortion resulting in the modification in Fe-O-Fe bond angle and octahedral tilt [13,20]. For x = 0.3 sample, an interesting modulation of magnetic order by the replacement of the diamagnetic cation Y^3+^ by the diamagnetic Bi^3+^ was observed. It can be correlated with the increase in structure distortion, due to the different ionic radii and tilt angle, together whith the effect of the Dzlayoshinski-Moriya interaction [31], providing a net magnetic moment. The inset in Figure 3 presented a hysteresis loops magnification for doping concentrations from x = 0 (un-doped) to 0.3. 

Further, in Figure 3, it was observed that a ferrimagnetic behavior with a low coercive field for x = 0.4, was attributed to the mixed magnetic orders of Y_3_Fe_5_O_12_, Bi_25_FeO_40_ and YFeO_3_. In addition, the increased specific magnetization at 18 kOe for x = 0.5 sample was correlated to the increased amount of the Y_3_Fe_5_O_12_ phase. In particular, the magnetic order of Y_3_Fe_5_O_12_ (YIG) prevailed in the magnetic hysteresis loops for x = 0.4 and x = 0.5 samples, due to its phase which was majoritarian in weight percent. However, specific magnetization at 18 kOe was lower as compared to pure Y_3_Fe_5_O_12_. Lower magnetic values can be explained due to Bi^3+^ cations which were into the Y_3_Fe_5_O_12_ crystal structure. Y_3_Fe_5_O_12_ has a cubic crystal structure with eight formula units and three sub-lattices, each one of these three sub-lattices are occupied by Y^3+^, and two Fe^3+^ cations; for 24c, 16a and 24d sites according to the Wyckoff notation [32,33]. Bi^3+^ cations promoted the formation of a new magnetic sub-lattice, which in turn caused a decrease in the magnetic order. Remanent magnetization slightly varied according to the Y_3_Fe_5_O_12_ content, from 0.06 to 3.24 emu/g for x = 0.3 and x = 0.5, respectively. However, the magnetic susceptively decreased as the bismuth content increased (from x = 0 to x = 0.3), and adscribed to the crystal distortion. The magnetic parameters obtained from magnetic hysteresis loops are summarized in Table 3.

**Figure 4:**
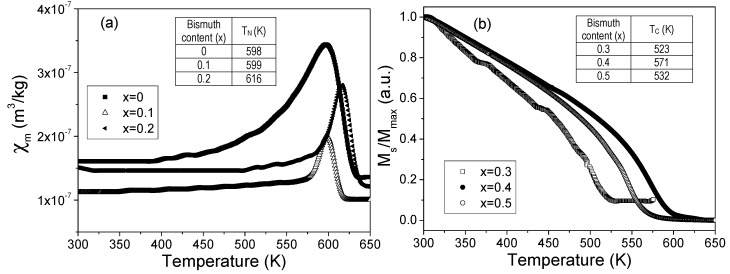
(**a**) Temperature dependence of magnetic susceptibility and, (**b**) magnetization of Y_1−x_Bi_x_FeO_3_ (0 ≤ x ≤ 0.5, Δx = 0.1) pressed and sinteredat 700 °C.

Finally, Figure 4 shows the effect of Bi^3+^ in the transition temperatures, Curie (FM to PM) and Néel (AF to PM), for the Y_1−x_Bi_x_FeO_3_ samples. As can be appreciated, a ferromagnetic to paramagnetic transitions were observed for 0.3 ≤ x ≤ 0.5 samples, while an antiferromagnetic to paramagnetic transitions were observed for 0 ≤ x ≤ 0.2 samples. The Néel temperature (Figure 4a) for un-doped samples was approximately 598 K, in good agreement with previous reports for this material [31]. For doped samples, the correlation between the increase of Bi^3+^ concentration and the increase in transition temperatures can be observed, in good agreement with Das et al. [34] and Yuan et al. [20]. This fact is attributed to the modification in the Fe-O-Fe bond angle shown in Table 2. Similar behavior was reported by Treves et al. [35] who proposed a link between average Fe^3+^-O^2−^-Fe^3+^ superexchange interaction with magnetic transition temperature, varying the temperature from 740 K to 623 K for LaFeO_3_ and LuFeO_3_, respectively.

Y_3_Fe_5_O_12_ has a Curie temperature approximately 553K [36], but some authors reported different values, adscribed to the presence of doping cations into the YIG structure, as Baños et al [37], who attributed this modifcation to a new magnetic interaction when Y^3+^ is substituted by Nd^3+^. The obtained Curie temperatures for x = 0.4 and 0.5 samples were in the range from 520 K to 571 K, around the value for pure Y_3_Fe_5_O_12_, the variations which have been attributed to the introduction of Bi^3+^ in the crystal structure and, the presence of secondary phases, as observed in the XRD patterns.

### 3.3. Dielectric Behavior 

**Figure 5:**
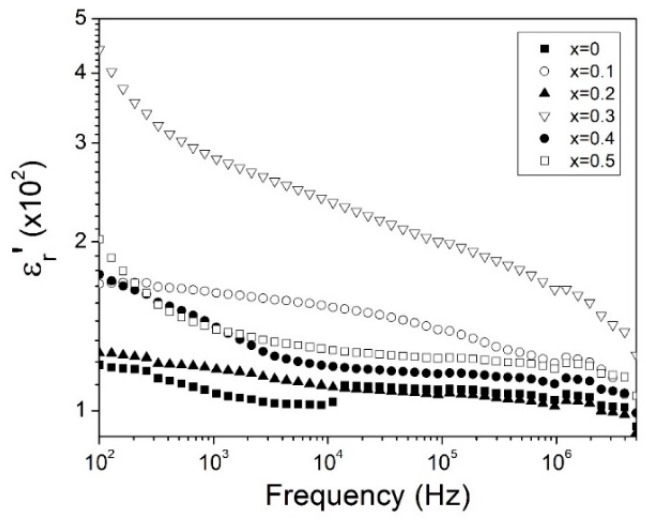
The real part of the complex relative permittivity (ε_r_’) at room temperature as a function of frequency of Bi^3+^ doped YFeO_3_ for 0 ≤ x ≤ 0.5, ∆x = 0.1, of pressed and sintered samples at 700 °C.

Impedance spectroscopy was used as an analytical tool to determine dielectric and electric properties of Bi^3+^ doped YFeO_3_. The frequency response for ferroelectric or dielectric materials usually is expressed in terms of relative complex permittivity (ε_r_*), which is composed for real (ε_r_’) and imaginary (ε_r_″) part, as observed from the relationship ε_r_*= ε_r_’ − jε_r_″ where $j=\sqrt{-1}$. ε’ and ε″ are correlated to polarization and energy losses of the system respectively [38]. Both real and imaginary relative permittivity parts can be calculated from impedance spectroscopy with the relation presented in Equations (5) and (6) [3]:

(5) $$\varepsilon_{r}^{\prime }=\frac{-Z^{\prime \prime }}{\omega C_{0}\left(Z^{\prime }{}^{2}+Z^{\prime \prime }{}^{2}\right)}$$

(6) $$\varepsilon_{r}^{\prime \prime }=\frac{Z^{\prime }}{\omega C_{0}\left(Z^{\prime }{}^{2}+Z^{\prime \prime }{}^{2}\right)}$$

where ω is angular frequency and C_0_ is the geometrical capacitance of the samples. The results of the real part of relative permittivity as a function of frequency are presented in Figure 5. For all samples, values of the real part of relative permittivity were in the range of ~4 × 10^2^ to ~1 × 10^2^ and tended to decrease with the increase of frequency. This can be attributed to space charge polarization produced by grain boundaries, and porosity, ascribed to the conformation process, which is in good agreement with the Maxwell-Wagner effect which predicts that at frequencies below 100 kHz, the principal contribution to the relative permittivity are charge accumulators like grain boundaries, defects and vacancies [39]. When the frequency is above the Maxwell-Wagner effect, it can be proposed that the dipoles responsible of polarization are not able to follow the oscillations of the field, thus an energy dissipation can be produced as evidenced by the reduction in ε_r_’ values [3,4]. The bismuth doped samples with x = 0.1 and 0.3 presented higher values of ε_r_’ than the un-doped sample (x = 0), while for x = 0.2 sample, the values were close to the un-doped sample. This increment may be explained by the increase in oxygen vacancies propitiated by Bi^3+^ cation in the samples. Oxygen vacancies are common as an inherent defect produced by heat treatment or doping in perovskite oxide structures [3,40]. 

This dielectric behaviors are very similar to those in the giant relative permittivity materials such as Ba(Fe_1/2_Nb_1/2_)O_3_, Sr(Fe_1/2_Nb_1/2_)O_3_, and Cu_3_CaTi_4_O_12_, however, relative permittivity is several orders lower than the giant relative permittivity materials. The samples with x = 0.4 and x = 0.5 showed a reduction in ε_r_’ values, in comparison with x = 0.3 sample, that can be correlated to the presence of Y_3_Fe_5_O_12_ and Bi_25_FeO_40_ as secondary phases.

**Figure 6:**
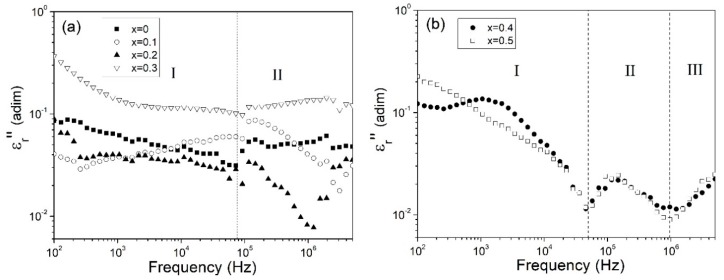
The imaginary part of the complex relative permittivity (ε’’) at room temperature as function of frequency of Y_1−x_Bi_x_FeO_3_ in the range of (**a**) 0 ≤ x ≤ 0.3 and (**b**) 0.4 ≤ x ≤ 0.5, of pressed and sintered samples at 700 °C.

The frequency dependence of the imaginary part of the complex relative permittivity (ε_r_’’) for doping samples in the ranges of 0 ≤ x ≤ 0.3 and 0.4 ≤ x ≤ 0.5 are shown in Figure 6a and b, respectively. For all samples ε_r_’’ showed a clear dependence with the frequency, having values which ranged from 3 × 10^−1^ to 8 × 10^−3^ for low and high frequencies respectively. For samples in the range 0 ≤ x ≤ 0.3, two regions can be observed; the first region at frequencies lower than 7 × 10^4^ Hz and the second one over the mentioned frequency. These regions are attributed to the two relaxations, as low temperature dielectric relaxation (LTDR) and high temperature dielectric relaxation (HTDR), following Hunpratub nomenclature for BiFeO_3_ [41], which can be observed at room temperature. On the one hand, the low temperature dielectric relaxation in YFeO_3_ ceramics originated from the electronic ferroelectricity by taking the possible mixed valence structure Fe^2+^/Fe^3+^ into account. On the other hand, the high temperature dielectric in YFeO_3_ ceramics has been related to the point defects, such as oxygen vacancies and the coexistence of Fe^2+^ and Fe^3+^, which have been generally formed during sintering in air. In the same way for samples in 0 ≤ x ≤ 0.3 range, the relaxation observed in the second region was more evident when bismuth concentration was increasing. 

For samples with Bi^3+^ doping concentrations above 0.4, three zones can be recognized for ε_r_’’, the first (I) at frequencies lower than 4 × 10^4^ Hz, the second (II) from 4 × 10^4^ to 1 × 10^6^ Hz, and the last one (III), at frequencies from 1 × 10^6^ to 5 × 10^6^ Hz. Each of these zones corresponded to a relaxation process. These relaxations showed an increase in comparison with samples with lower bismuth which may be attributed to the presence of secondary phases, especially bismuth. As can be observed, the bismuth concentration directly correlated to changes in the relaxation process in all samples. It has been demonstrated that doping produces the appearance of new dielectric relaxation that can be associated at oxygen vacancies [11,17]. Liberated electrons from oxygen vacancies can be taken by Fe^3+^ ions and change their oxidation state to Fe^2+^ ions [42]. The hopping of the electrons through the Fe^2+^-O^2−^-Fe^3+^ bond chains, which is equivalent to the dipole reorientation under ac fields, can also give rise to the dielectric relaxation in Y_1−x_Bi_x_FeO_3_ samples.

### 3.4. Conductivity and Leakage Current

**Figure 7:**
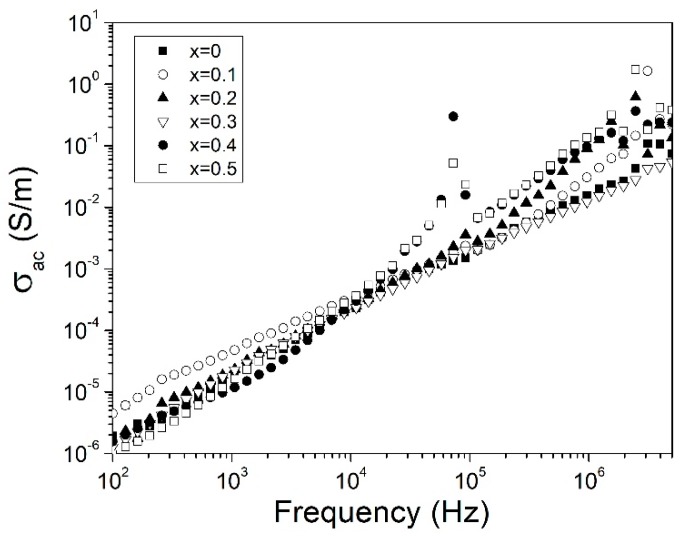
Alternating current conductivity (σ_AC_) at room temperature of Y_1−x_Bi_x_FeO_3_ varying *x* from 0 to 0.5, of pellets sintered at 700 °C.

In order to determine the effect of alternating current conductivity (σ_ac_) in dielectric properties at room temperature of selected samples, σ_ac_ was calculated employing the relation shown in Equation (7):

(7) $$\sigma_{ac}=\frac{Z^{\prime }}{Z^{\prime }{}^{2}+Z^{\prime \prime }{}^{2}}\frac{d}{A}$$

where Z’ and Z″ correspond to the real and imaginary part of complex impedance, *A* is the cross-sectional area and *d* is the thickness of the sample. Frequency dependence of σ_ac_ is presented in Figure 7. For all doping concentrations, the σ_ac_ increased as the frequency was augmented. The increased conductivity can be attributed to the transition from electronic to ionic conduction [39]. Similar behavior has been reported for other ferrites, where grain boundaries acted like insulators between grains reducing electron mobility [43,44]. The increased frequency reduced the effect of grain boundaries, which increased the conductivity. Also, the conductivity increased when the bismuth doping concentration increased. This behavior can be attributed to an increase in oxygen vacancies, besides the grain size. Oxygen vacancies can be produced in many ways as sintering at high temperatures or, destabilization in the structure. Changes in the crystal structure, especially referred to the Fe-O bond, produced oxygen vacancies which caused an increase in Fe^2+^ and in consequence, an increase in hopping charge carriers and conductivity [45]. Additionally to oxygen vacancies, increased grains size propitiated a reduction of grain boundaries in the doped samples, and then reduced physical barriers that inhibited electron mobility, which increased the conductivity. Finally, as can be seen in Figure 7, the alternating current conductivity (σ_ac_), for all evaluated samples, were in the range of the semiconductor materials from ~1 × 10^−6^ to ~4 × 10^−1^ S/cm, for 10^2^ and 5 × 10^6^ Hz, respectively.

**Figure 8:**
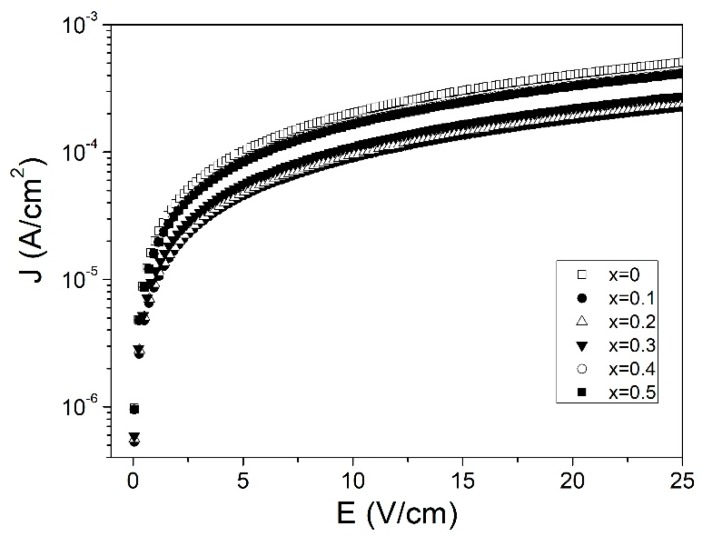
The leakage current density as function of electric field at room temperature of Y_1−x_Bi_x_FeO_3_ with 0 ≤ x ≤ 0.5 samples sintered at 700 °C.

Leakage current dependence as a function of applied voltage of positive bias was measured for the selected samples. Figure 8 presents the leakage current density (J) versus electric field (E) for Bi^3+^ doped samples at different concentrations, 0 ≤ x ≤ 0.5. For all the samples, the behavior of current density showed a very sharp slope at low field values followed by a less steep slope, which was not able to reach saturation due to the limitation of the applied field from the LCR meter. The leakage current in ceramics samples has been affected by factors as microstructure, vacancy oxygen, and grain size and their boundaries as they provided conduction pathways [46,47]. Lubomirsky found that contact produced by the contact between grains of very different sizes lead to exchanging between them, known as heterosize charging [48]. Reddy et al. reported that leakage current was affected by non-uniform grain structures and porous structures, which were the product of heterosize charging [49]. Bismuth doping reduced the leakage current, in comparison with the un-doped sample, which showed the lowest value for x = 0.1. The leakage current reduction can be correlated to increased crystallinity and reduced porosity as has been observed previously in XRD and SEM respectively. As can be observed from the SEM micrographs, Bi^3+^ reduced porosity and increased grain size providing a more homogeneous structure which reduced the leakage current by a reduction in heterosize charging. Hasan et al. and Das et al. have reported that the substitution of a smaller cation for a larger one produced a reduction in movable charge density and reduced the leakage current [7,34]. Similar behavior may have occurred when the Y^3+^ cation was substituted for a larger Bi^3+^ cation, which reduced movable charges in comparison with the un-doped sample. In addition, the increased leakage current density when bismuth contained > 0.3 may be attributed to the coexistence of multiple phases which produced an increment of heterosize charging due to the coexistence of different grains morphologies and grain boundaries.

## 4. Conclusions

Ceramic samples Y_1−x_Bi_x_FeO_3_ at doping concentrations 0 ≤ x ≤ 0.5, were synthesized by means of high-energy ball milling followed by pressing and sintered at 700 °C. Their magnetic and dielectric properties were evaluated. The Bi^3+^ doping concentrations, below x = 0.3, affected the lattice parameters but did not produce crystal structure transitions, keeping the orthorhombic structure of yttrium orthoferrite. An increase in the octahedral angle tilt was observed when the addition of Bi^3+^ was increased, providing a path for change for both magnetic and dielectric behavior. A weak magnetic moment was found for sample with x = 0.3 which was attributed to both the crystal distortion and the Dzyaloshinskii-Moriya interaction. For doping concentrations of Bi^3+^ higher than x = 0.3, crystal structure transformations were produced from yttrium orthoferrite to Y_3_Fe_5_O_12_ and Bi_25_FeO_40_. The samples mainly composed with YIG possessed a significant ferromagnetic order, but with saturation magnetization, the amount lowered due to the paramagnetic contribution of sillenite as a secondary phase. The dielectric behavior presented great similitude with the giant dielectric constant material. However, the magnitude order was lower than these materials. In order to understand this phenomenon, it is necessary to undertake an investigation in relation to temperature and dielectric behavior. The Bi^3+^ and sintering process promoted the formation of oxygen vacancies and increased conductivity. The Bi^3+^ reduced the leakage current of yttrium orthoferrite due to an increase in grain, crystallite size and a reduction in porosity.

## Figures and Tables

**Table 1:** Rietveld analysis of the XRD patterns to Y_1−x_Bi_x_FeO_3_ pellets of all compositions.

Level Doped (*x*)	Phase Space Group	% Phase	Lattice Parameters (Å)			Crystallite Size (Å)	µε (×10^−4^)	χ^2^	R_wp_
			a	b	c				
0	YFeO_3_ *Pnma*	100 ± 0.7230	5.2683 ± 0.0005	5.5510 ± 0.0004	7.5764 ± 0.0005	911.11 ± 56.58	41.41 ± 0.0001	1.03	12.04
0.1	YFeO_3_ *Pnma*	100 ± 0.6692	5.2714 ± 0.0004	5.5664 ± 0.0004	7.5845 ± 0.0005	929.39 ± 31.46	16.24 ± 0.0006	1.16	17.49
0.2	YFeO_3_ *Pnma*	100 ± 0.5242	5.2974 ± 0.0002	5.5854 ± 0.0002	7.6224 ± 0.0003	1270.91 ± 31.25	11.77 ± 0.0001	1.20	16.34
0.3	YFeO_3_ *Pnma*	100 ± 0.9614	5.3117 ± 0.0002	5.5879 ± 0.0003	7.6411 ± 0.0004	1492.36 ± 55.61	10.27 ± 0.0004	1.23	16.81
0.4	YFeO_3_ *Pnma*	15.97 ± 0.3432	5.3139 ± 0.0010	5.5944 ± 0.0010	7.6461 ± 0.0009	952.63 ± 22.87	7.23 ± 0.0002	1.24	24.59
	Y_3_Fe_5_O_12_ *I-a3d*	73.67 ± 0.2156	12.3835 ± 0.0003	-	-	1819.16 ± 126.78	8.68 ± 0.0008		
	Bi_25_FeO_40_ *I-23*	10.36 ± 0.5221	10.0856 ± 0.0014	-	-	999.91 ± 257.04	29.35 ± 0.0003		
0.5	Y_3_Fe_5_O_12_ *I-a3d*	86.68 ± 0.3264	12.3931 ± 0.0003	-	-	2768.51 ± 293.71	13.64 ± 0.0004	1.27	25.25
	Bi_25_FeO_40_ *I-23*	13.32 ± 0.5678	10.1573 ± 0.0003	-	-	1625.94 ± 107.13	4.59 ± 0.0001		

**Table 2:** Atomic positions (x, y and z), Fe-O-Fe bond angles (θ1 and θ2), tilt angles (φ_1_ and φ_2_) and tolerance factor of YFeO_3_ phase of samples sintered at 700 °C.

Bismuth Content (x)	Elem.	Occ.	Atomic Positions			Bond Angle (Degree)		Tilt Angle (Degree)		
			*x*	*y*	*z*	θ1	θ2	φ_1_	φ_2_	*t (adim)*
0	Y	1	0.06399 ± 0.0004	0.2500 ± 0.0000	−0.0174 ± 0.0007	155.16 ± 0.10	142.12 ± 0.80	15.095	23.424	0.834
	Fe	1	0.0000	0.0000	0.5000					
	O1	1	0.4861 ± 0.0026	0.2500 ± 0.0000	0.0779 ± 0.0028					
	O2	1	−0.2975 ± 0.0022	−0.0701 ± 0.0017	0.2986 ± 0.0027					
0.1	Y	0.9	0.0625 ± 0.0005	0.2500 ± 0.0000	−0.0118 ± 0.0010	152.51 ± 0.10	139.29 ± 0.80	16.667	25.215	0.839
	Bi	0.1	0.0625 ± 0.0005	0.2500 ± 0.0000	−0.0118 ± 0.0010					
	Fe	1	0.0000	0.0000	0.5000					
	O1	1	0.4792 ± 0.0035	0.2500 ± 0.0000	0.0852 ± 0.0036					
	O2	1	−0.2967 ± 0.0029	−0.0815 ± 0.0018	0.2951 ± 0.0029					
0.2	Y	0.8	0.0587 ± 0.0004	0.2500 ± 0.0000	−0.0102 ± 0.0007	149.71 ± 0.10	138.54 ± 0.80	18.340	25.691	0.843
	Bi	0.2	0.0587 ± 0.0004	0.2500 ± 0.0000	−0.0102 ± 0.0007					
	Fe	1	0.0000	0.0000	0.5000					
	O1	1	0.4735 ± 0.0024	0.2500 ± 0.0000	0.09329 ± 0.0024					
	O2	1	−0.2983 ± 0.0023	−0.0813 ± 0.0014	0.2973 ± 0.0023					
0.3	Y	0.7	0.0538 ± 0.0004	0.2500 ± 0.0000	−0.0062 ± 0.0007	149.13 ± 0.10	136.63 ± 0.80	18.683	26.907	0.848
	Bi	0.3	0.0538 ± 0.0004	0.2500 ± 0.0000	−0.0062 ± 0.0007					
	Fe	1	0.0000 ± 0.0000	0.0000 ± 0.0000	0.5000 ± 0.0000					
	O1	1	0.4743 ± 0.0025	0.2500 ± 0.0000	0.0955 ± 0.0023					
	O2	1	−0.2935 ± 0.0024	−0.0914 ± 0.0013	0.2881 ± 0.0024					

**Table 3:** Specific magnetization (M_s_), mass magnetic susceptibility (χ_m_), remanent magnetization (M_r_) and coercive field (H_c_) for Bi doped YFeO_3_.

Bi^3+^ Doping Concentration (x)	M_s_ at 18 kOe (emu/g)	M_r_ (emu/g)	H_c_ (Oe)	χ_m_ (×10^−7^ m^3^/kg)
0	0.34	0.02-	710-	2.34
0.1	0.21	-	-	1.66
0.2	0.22	-	-	1.54
0.3	0.38	0.06	495	-
0.4	4.42	1.18	251	-
0.5	14.72	3.24	202	-
